# Trends in Tuberculosis Incidence and Treatment Outcomes in Kazakhstan: A Decade of Observational Data

**DOI:** 10.3390/tropicalmed11030075

**Published:** 2026-03-06

**Authors:** Galymzhan Ryskulov, Malik Adenov, Maira Zhaparkulova, Alibek Bissembayev, Gulnar Rakhimbekova, Dariga Tanabayeva, Shynar Tanabayeva, Ildar Fakhradiyev, Marat Shoranov

**Affiliations:** 1 Department of Medicine, National Scientific Center of Phthisiopulmonology of the Republic of Kazakhstan, Almaty 050010, Kazakhstan; 2 School of IT and Engineering, Kazakh-British Technical University, Almaty 050000, Kazakhstan; 3 Department of Medicine, Astana Medical University, Astana 010000, Kazakhstan; 4 Department of Cellular and Physiological Sciences, Faculty of Science, University of British Colombia, Vancouver, BC V6T 1Z4, Canada; 5 Department of Medicine, S.D. Asfendiyarov Kazakh National Medical University, Almaty 050000, Kazakhstan; 6 College of Medicine, Korea University, Seoul 02841, Republic of Korea

**Keywords:** tuberculosis, incidence, treatment outcome, risk factors, retrospective studies, Kazakhstan

## Abstract

**Background**: Tuberculosis (TB) remains a major public health concern globally, despite sustained declines in incidence in many countries. Kazakhstan has implemented long-term national TB control strategies; however, comprehensive nationwide analyses integrating temporal trends, demographic patterns, and treatment outcomes over the past decade remain limited. **Methods**: A nationwide retrospective registry-based analysis of programmatic TB treatment episodes was conducted using anonymized data from the national tuberculosis registry of the Ministry of Health of Kazakhstan. All registered TB cases from 1 January 2014 to 31 December 2023 were included. Treatment outcome was analyzed as the final end-of-episode programmatic status (favorable vs. unfavorable). Because the anonymized extract did not contain complete patient-level dates required to derive person-time (treatment initiation and event dates), time-to-event models were not applied; instead, factors associated with unfavorable end-of-treatment outcomes were assessed using multivariable logistic regression and reported as adjusted odds ratios (aORs) with 95% CIs. Unfavorable treatment outcomes were defined as death, treatment failure, loss to follow-up, and not evaluated or not recorded outcome, according to the national TB program outcome definitions. **Results**: A total of 93,985 TB cases were analyzed. The number of registered cases declined from 16,391 in 2014 to 6548 in 2023, corresponding to a cumulative reduction of 60.1% and an AAPC of −9.7% per year. TB incidence decreased in both sexes, although rates remained consistently higher among men. Over time, the peak incidence shifted toward older age groups, particularly among men. The proportion of new cases increased to 80.1% by 2023, while relapses and treatment failures declined. In multivariable analysis, unfavorable treatment outcomes were independently associated with male sex (aOR 1.25), older age, relapse, treatment after interruption, prior treatment failure, smear-positive disease (aOR 1.60), combined pulmonary and extrapulmonary involvement, and disseminated TB (ICD-10 A19). The risk of unfavorable outcomes increased during 2020–2021 and declined in 2022–2023. **Conclusions**: Kazakhstan has achieved a substantial and sustained reduction in TB incidence over the past decade. Nevertheless, marked demographic and clinical disparities persist, particularly among men, older patients, smear-positive cases, and individuals with prior or interrupted treatment. Targeted interventions focused on these high-risk groups may further improve treatment outcomes and support continued progress toward TB control.

## 1. Introduction

Tuberculosis (TB) is an infectious disease caused by *Mycobacterium tuberculosis* that adversely affects both the physical health and psychosocial well-being of patients [1,2]. Globally, tuberculosis remains a major public health challenge. In 2023, 8.2 million new TB cases were reported worldwide—an increase from 7.5 million in 2022 and 7.1 million in 2019, and considerably higher than the 5.8 million and 6.4 million cases reported in 2020 and 2021, respectively [3].

A substantial proportion of these newly diagnosed cases in 2022 and 2023 likely represent infections that developed in previous years but whose diagnosis and treatment were delayed due to the COVID-19 pandemic, as noted in the WHO’s Global Tuberculosis Report 2024 [3].

However, these figures vary significantly by region. In 2019, the WHO regions of South-East Asia, Africa, and the Western Pacific accounted for approximately 44%, 25%, and 18% of global TB cases, respectively. In contrast, the Eastern Mediterranean, the Americas, and Europe reported much lower shares, ranging from 2% to 8%. Overall, two-thirds of TB cases were concentrated in just eight high-burden countries [4].

Although global TB mortality continued to decline in 2023, reinforcing the downward trend that began in 2022 after a surge during the height of the COVID-19 pandemic in 2020 and 2021, statistics suggest that TB may once again be the leading cause of death from infectious diseases worldwide, overtaking COVID-19 [5].

Kazakhstan, the largest country in Central Asia, launched its National Tuberculosis Control Program in 1998 under the Ministry of Health. This program, which emphasizes early detection and treatment, incorporates directly observed short-course therapy (DOTS) and has led to significant reductions in TB incidence [6]. Despite these efforts, tuberculosis remains an ongoing public health concern in Kazakhstan, with continued case notifications and substantial heterogeneity across regions and population groups [7]. However, the current body of literature remains fragmented and often focuses either on specific regions, selected patient cohorts, or limited time windows. Recent analyses based on large-scale administrative and surveillance data have described declining TB incidence prior to and during the pandemic years, while also highlighting persistent adverse outcomes in certain demographic and clinical groups [8]. Other studies have documented pandemic-related disruptions to TB services and their association with less favorable treatment outcomes in specific settings [9], as well as regional differences in TB incidence and mortality patterns before, during, and after COVID-19 [10]. Nevertheless, a consolidated nationwide assessment spanning the past decade that jointly examines TB incidence and treatment outcomes stratified by demographic and clinical characteristics remains limited. This gap constrains the evidence base for targeted public health interventions and resource allocation.

The objective of this research is not only to assess the prevalence of TB but also to identify clinical patterns and outcomes across different demographic groups. This study represents a comprehensive analysis of nationwide trends in TB incidence and treatment outcomes in Kazakhstan over the past decade. It also provides a foundation for the development of more targeted public health interventions, particularly for vulnerable populations with worse outcomes and a higher risk of unfavorable outcomes.

## 2. Materials and Methods

### 2.1. Study Design

A nationwide retrospective registry-based observational study was conducted using national TB surveillance data. The unit of analysis was a registered TB treatment episode, and treatment outcome was assessed as the final programmatic status recorded for that episode. The analysis covered a ten-year period from 1 January 2014 to 31 December 2023 and included all registered tuberculosis cases in the Republic of Kazakhstan.

### 2.2. Study Population and Data Source

We analyzed all notified tuberculosis cases recorded in the National TB Register for the study period. In Kazakhstan, registration of infectious diseases is mandatory at the place of detection and applies to all healthcare subjects, including the public and non-public sectors and private medical practice, with national reporting procedures defined by the Ministry of Health. The National TB Register is the national information system used to collect and manage patient-level TB treatment and laboratory data.

### 2.3. Inclusion and Exclusion Criteria

The study included patients with a confirmed diagnosis of tuberculosis corresponding to ICD-10 codes A15–A19 (International Classification of Diseases, 10th revision). The following were excluded from the analysis: duplicate records and cases with missing data on key variables (sex, age, case type, or treatment outcome).

### 2.4. Study Variables

Demographic and clinical-epidemiological characteristics of patients were analyzed. Demographic variables included sex (male, female) and age at the time of case registration. For analytical purposes, age was categorized into the following groups: 0–14, 15–24, 25–34, 35–44, 45–54, 55–64, and ≥65 years.

Clinical and epidemiological variables included case type (new case, relapse, treatment failure, treatment after interruption), disease localization (pulmonary, extrapulmonary, combined pulmonary and extrapulmonary tuberculosis), ICD-10 diagnosis (A15–A19), sputum smear microscopy result (positive, negative), treatment outcome (cured, treatment completed, treatment failure, death, lost to follow-up, outcome not evaluated), and year of case registration (2014–2023).

For analysis of factors associated with treatment outcome, outcomes were additionally dichotomized into favorable (cured, treatment completed) and unfavorable (treatment failure, death, lost to follow-up, outcome not evaluated).

The analysis focused on the final recorded treatment outcome for each episode. Dates of treatment initiation and the exact timing of unfavorable events were not consistently available in the anonymized dataset used for this study; therefore, person-time could not be derived.

### 2.5. Population Denominators

Annual mid-year population estimates for the Republic of Kazakhstan stratified by sex and age group for 2014–2023 were obtained from the Bureau of National Statistics. Tuberculosis incidence rates were calculated as the number of notified cases divided by the corresponding annual mid-year population and expressed per 100,000 population. Age-standardized incidence rates were computed using direct standardization by calculating age-specific rates in ten missing age groups, applying the selected standard population weights, and summing the weighted rates to obtain the age-standardized rate per 100,000 population. The average annual percent change was estimated using a log-linear model with the natural logarithm of the age-standardized incidence rate as the dependent variable and calendar year as the independent variable.

### 2.6. Statistical Analysis

Statistical data processing was performed using IBM SPSS Statistics version 27.0. All registered tuberculosis cases for 2014–2023 were included in the analysis. Incidence was described using absolute and relative indicators as well as rates per 100,000 population; cumulative reduction relative to 2014 and the average annual percent change (AAPC) were calculated. Distributions of categorical variables were compared using Pearson’s χ^2^ test. Factors associated with an unfavorable treatment outcome were assessed using multivariable logistic regression with a binary dependent variable (unfavorable vs. favorable outcome). Accordingly, the regression model estimates associations with the probability of an unfavorable end-of-episode outcome rather than the hazard of event occurrence over time. To quantify the decline in annual registered TB case counts over time, we additionally applied count regression models. Annual case counts were modeled using a generalized linear model with a Poisson distribution and log link, with calendar year entered as a continuous predictor. Overdispersion was assessed using the Pearson chi-square to degrees-of-freedom ratio; when present, robust (sandwich) standard errors and, as a sensitivity analysis, negative binomial regression were considered. Model estimates are reported as incidence rate ratios (IRR) per one-year increase with 95% confidence intervals; the average annual percent change was derived as (1 − IRR) × 100. The model included sex, age group, case type, disease localization, sputum smear microscopy result, ICD-10 diagnosis, and calendar period; categorical variables were entered using indicator coding, and reference categories were defined a priori. Results are presented as adjusted odds ratios (aORs) with 95% confidence intervals. Statistical significance was assessed at *p* < 0.05. To account for potential within-cluster correlation in this nationwide dataset, we calculated Huber–White robust standard errors clustered at the region of registration level (G = 20 clusters; median cluster size 4650 treatment episodes; range 720–12,480). We report cluster-robust 95% confidence intervals and *p*-values. As a sensitivity analysis, we additionally fitted a multilevel logistic regression model with a random intercept for region; effect estimates were consistent with the primary clustered-inference analysis.

## 3. Results

### Temporal and Gender Trends in Tuberculosis Incidence

In 2014–2023, the Republic of Kazakhstan recorded a pronounced and sustained decline in registered tuberculosis. The total number of cases decreased from 16,391 in 2014 to 6548 in 2023, corresponding to a cumulative reduction of 60.1% compared with the baseline level. Poisson count regression supported a consistent downward trend in annual registered TB case counts. Each additional calendar year was associated with an IRR of 0.895 (95% CI 0.872–0.919), corresponding to an average annual reduction of 10.5% (95% CI 8.1–12.8). The model-implied reduction from 2014 to 2023 was 63.1% (95% CI 53.4–70.8), which was consistent with the observed cumulative reduction.

The average annual percent change (AAPC) over the analyzed period was −9.7% per year. The decline was systemic in nature and was observed among both men and women. The average annual rates of decrease were comparable in the two groups and amounted to 9.6% in men and 9.8% in women, with no statistically significant differences between sexes. The most pronounced reduction was registered in 2014–2020, after which stabilization of the indicators was observed in 2021–2023 at a level of about 40% of the 2014 values (Table 1). 

**Figure 1:**
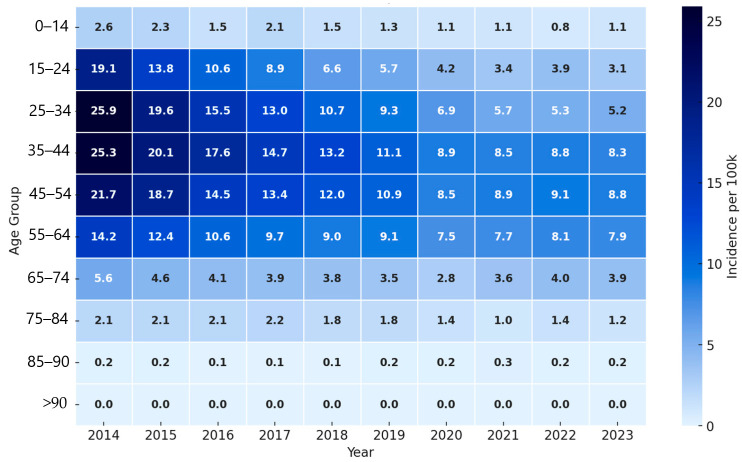
Age-specific incidence of tuberculosis among men per 100,000 population, by age group and calendar year, 2014–2023.

Among men, the highest age-specific notification rates per 100,000 population in 2014 and 2015 were observed in the 25–34 age group, reaching 25.9 and 19.6, respectively, based on registered tuberculosis cases and annual population denominators from the Bureau of National Statistics (Figure 1). Starting from 2016, the peak rate gradually shifted to older age groups. During 2016 to 2020, the highest rates were recorded in the 35–44 age group, and since 2021 in the 45–54 age group.

**Figure 2:**
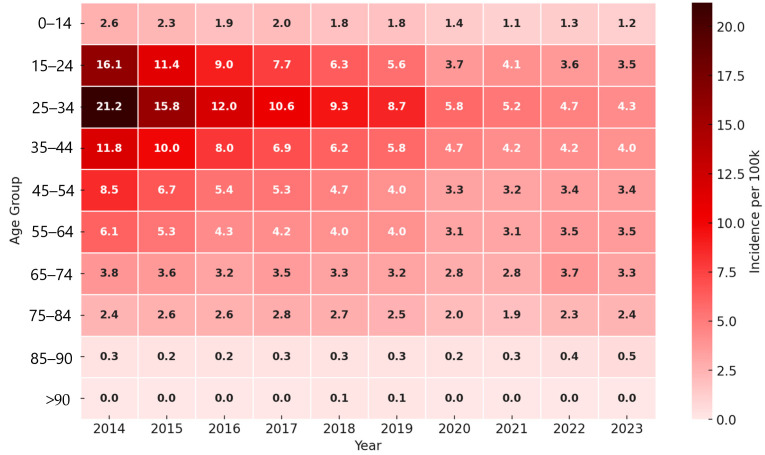
Age-specific incidence of tuberculosis among women per 100,000 population, by age group and calendar year, 2014–2023.

Among women, the highest age-specific notification rate was also consistently observed in the 25–34 age group, particularly in 2014 at 21.2 per 100,000 population (Figure 2). Compared with men, rates among women were lower in all age groups throughout the observation period.

Overall, age-specific notification rates decreased across all age groups in both sexes. Pronounced gender and age differences persisted. Men consistently had higher rates than women, especially in the 25–54 age group.

The structure of tuberculosis cases in the Republic of Kazakhstan for 2014–2023, distributed by case type according to sex, age group, and year of registration (Table 2), showed that new cases accounted for 73.2% (n = 68,769), relapses 24.6% (n = 23,075), treatment failure 1.0% (n = 944), and treatment after interruption 1.3% (n = 1197). The distribution of case types differed significantly by sex (χ^2^ = 633.8, df = 3, *p* < 0.001).

Among women, the proportion of relapses was 26.8%, and the proportion of cases treated after interruption was 1.7%; among men, these were 21.2% and 0.7%, respectively, whereas the proportion of new cases was higher in men (77.3%) than in women (70.4%). Marked age-related differences in the structure of case types were identified (χ^2^ = 6630.5, df = 27, *p* < 0.001). In the 0–14 age group, new cases accounted for 97.7%, relapses 2.0%, treatment failure 0.3%, and treatment after interruption 0.1%. With increasing age, the proportion of relapses increased and reached 38.4% in the group aged 65 years and older, while the proportion of new cases decreased to 59.6%. The distribution of case types also differed by year of registration (χ^2^ = 1768.5, df = 27, *p* < 0.001). The proportion of new cases increased from 65.7% in 2014 to 80.1% in 2023, whereas the proportion of relapses decreased from 31.5% to 19.3%, and the proportion of treatment failures declined from 1.3% to less than 0.1%.

Inference for the regression model was based on robust standard errors clustered by region of registration. In the multivariable logistic regression (Table 3 and Table S1), adjusted for sex, age, case type, disease site, bacterioexcretion, ICD-10 diagnosis, and calendar period, an unfavorable tuberculosis treatment outcome was statistically significantly associated with a number of demographic and clinical factors. Male sex was associated with an increased risk of an unfavorable outcome (aOR = 1.25; 95% CI 1.17–1.33; *p* < 0.001). A pronounced age gradient was observed: compared with the 25–34-year group, the risk was lower among children aged 0–14 years (aOR = 0.55; 95% CI 0.43–0.70; *p* < 0.001) and individuals aged 15–24 years (aOR = 0.80; 95% CI 0.73–0.88; *p* < 0.001), but increased consistently in older age groups, reaching a maximum among patients aged ≥75 years (aOR = 2.10; 95% CI 1.76–2.51; *p* < 0.001).

Case type had one of the strongest effects on treatment outcomes: relapses (aOR = 1.90; 95% CI 1.73–2.08; *p* < 0.001), cases after treatment interruption (aOR = 2.60; 95% CI 2.20–3.07; *p* < 0.001), and the treatment failure category (aOR = 2.10; 95% CI 1.60–2.76; *p* < 0.001) had a substantially higher risk of an unfavorable outcome compared with new cases. Extrapulmonary tuberculosis was associated with a more favorable prognosis (aOR = 0.70; 95% CI 0.61–0.80; *p* < 0.001), whereas combined pulmonary and extrapulmonary involvement was characterized by an increased risk of an unfavorable outcome (aOR = 1.40; 95% CI 1.12–1.75; *p* = 0.003).

Positive smear microscopy was an independent predictor of an unfavorable outcome (aOR = 1.60; 95% CI 1.48–1.73; *p* < 0.001). Compared with diagnosis A16, codes A15 (aOR = 1.35; 95% CI 1.23–1.48; *p* < 0.001) and A19 (aOR = 2.05; 95% CI 1.76–2.39; *p* < 0.001) were associated with poorer outcomes, whereas A18 was characterized by a reduced risk (aOR = 0.85; 95% CI 0.74–0.98; *p* = 0.025). The association for A17 vs. A16 became borderline after clustered inference (aOR = 1.80; 95% CI 0.97–3.35; *p* = 0.064). The temporal factor was also significant: in 2020–2021, the risk of an unfavorable outcome was higher compared with 2014–2016 (aOR = 1.15; 95% CI 1.04–1.27; *p* = 0.006), followed by a decrease in 2022–2023 (aOR = 0.90; 95% CI 0.81–1.00; *p* = 0.044).

## 4. Discussion

The present nationwide retrospective registry-based study provides a comprehensive overview of tuberculosis epidemiology and treatment outcomes in Kazakhstan over a ten-year period (2014–2023). The analysis demonstrates a pronounced and sustained decline in registered TB incidence, accompanied by persistent demographic and clinical heterogeneity in disease patterns and outcomes. Because this analysis was conducted at the national level, treatment outcomes may be correlated within geographic units. To avoid underestimating uncertainty, we based inference in the treatment-outcome model on cluster-robust standard errors at the region of registration level (G = 20 clusters). This approach led to modestly wider confidence intervals, without changing point estimates; the main conclusions were unchanged, although the association for A17 vs. A16 became borderline after clustering.

Over the study period, TB incidence in Kazakhstan decreased by 60.1% compared with 2014, with an average annual percent change of −9.7%. This decline was most pronounced between 2014 and 2020, followed by stabilization in 2021–2023.

Such dynamics are consistent with the long-term implementation of national TB control measures, including DOTS expansion and standardized treatment, although concurrent changes in diagnostic practices, reporting completeness, and access to care may also have influenced registered incidence.

At the same time, the plateau observed after 2020 coincides with the COVID-19 pandemic [10,11], suggesting that reduced access to healthcare services and delayed diagnosis may have temporarily limited further reductions in registered incidence [12].

Despite comparable rates of decline in both sexes, TB incidence remained consistently higher among men throughout the study period. Importantly, male sex was independently associated with a higher risk of unfavorable treatment outcomes (aOR 1.25).

This association suggests that sex differences persist in observed programmatic treatment outcomes. However, unmeasured factors such as drug resistance, HIV co-infection, and comorbidities may partly contribute to this pattern and could confound the estimated effect size [13].

On balance, this pattern is likely multifactorial and may reflect delayed healthcare seeking, higher prevalence of behavioral risk factors, and lower treatment adherence among men [14]. Notably, the persistence of this effect after multivariable adjustment suggests that male sex itself remains a marker of vulnerability within TB control efforts [15].

A clear age-related shift in TB incidence was observed, particularly among men, with the peak moving from younger (25–34 years) to older age groups (45–54 years) over time. This pattern is consistent with an aging TB cohort and may reflect reactivation of latent infection rather than ongoing transmission among younger populations [16].

Age also played a critical role in treatment outcomes. While children and young adults had a lower risk of unfavorable outcomes, a strong and progressive age gradient was observed from 35 years onward, reaching a twofold increase in patients aged ≥75 years. This likely reflects the cumulative impact of comorbidities, immunosenescence, and challenges in treatment tolerance and adherence in older patients [17].

The structure of TB cases changed substantially over the decade. The proportion of new cases increased to more than 8 0% by 2023, while relapses and treatment failures declined markedly. This shift suggests improved treatment effectiveness and follow-up at the population level [18].

However, relapse, treatment after interruption, and prior treatment failure emerged as the strongest predictors of unfavorable outcomes in multivariable analysis. Patients treated after interruption had the highest risk (aOR 2.60), followed by those with treatment failure and relapse. These findings underscore that, despite overall programmatic success, a relatively small subgroup of patients with disrupted or repeated treatment courses contributes disproportionately to poor outcomes and likely to ongoing transmission and drug resistance [19].

Several clinical characteristics independently predicted treatment outcomes. Smear-positive disease was strongly associated with unfavorable outcomes (aOR 1.60), highlighting the prognostic importance of bacterial burden at diagnosis [20].

Localization also played a significant role: extrapulmonary TB was associated with more favorable outcomes, whereas combined pulmonary and extrapulmonary involvement carried an increased risk [21]. This likely reflects greater disease severity and diagnostic complexity in disseminated forms [22].

Differences by ICD-10 category further supported this interpretation. Patients coded as A19 (miliary or disseminated TB) had the highest risk of poor outcomes, whereas A18 diagnoses were associated with a more favorable prognosis. These findings suggest that routine diagnostic coding can serve as a useful proxy for disease severity in large registry-based analyses [8].

The calendar period 2020–2021 was independently associated with an increased risk of unfavorable outcomes, followed by a significant improvement in 2022–2023. This temporal pattern supports the hypothesis that the COVID-19 pandemic disrupted TB services, affecting continuity of care and treatment supervision. The subsequent reduction in risk likely reflects recovery and adaptation of TB services in the post-pandemic period [23].

Taken together, these findings indicate that Kazakhstan has achieved substantial progress in reducing TB incidence, yet important challenges remain. Men, older adults, smear-positive patients, and those with prior or interrupted treatment represent priority groups for targeted interventions. Strengthening adherence support, early detection of relapse, and focused follow-up of high-risk patients may yield further improvements in treatment outcomes [24].

## 5. Conclusions

From 2014 to 2023, Kazakhstan achieved a substantial decline in registered TB cases from 16,391 to 6548, corresponding to a 60.1% reduction and an average annual percent change of −9.7%, while rates remained consistently higher among men, and the peak burden shifted toward older age groups. Over the same period, the proportion of new cases increased to 80.1% by 2023, whereas the shares of relapses and treatment failures decreased between 2014 and 2023. Unfavorable treatment outcomes were independently associated with male sex, older age, relapse, treatment after interruption, prior treatment failure, smear-positive disease, combined pulmonary and extrapulmonary involvement, and disseminated TB (A19), with an increased risk in 2020 to 2021, followed by improvement in 2022 to 2023. These findings may help inform programmatic prioritization by identifying patient groups with higher odds of unfavorable end of treatment outcomes. Targeted adherence support and follow-up for retreatment categories, smear-positive disease, and disseminated or combined forms, especially among men and older adults, are reasonable areas for focus, while the expected impact should be interpreted cautiously given the lack of adjustment for drug resistance, HIV, and major comorbidities.

## 6. Study Limitations

This study relied on routinely collected registry data, and the anonymized extract did not include drug resistance status, HIV co-infection, or key comorbidities such as diabetes, as well as detailed socioeconomic variables. Given the historically high burden of MDR TB in Kazakhstan, the inability to adjust for resistance and HIV status is a major limitation and may lead to residual confounding because these factors are strong predictors of both treatment outcomes and several clinical or programmatic characteristics included in the models. Therefore, the reported associations should be interpreted as programmatic correlations rather than causal effects, and the magnitude of some adjusted odds ratios may be biased in either direction. Nevertheless, the nationwide scope, long observation period, and robust multivariable analysis provide a solid basis for population-level interpretation. In addition, the anonymized registry extract did not include complete dates needed to model time-to-event outcomes (treatment start and event dates), precluding survival analysis and limiting inference to end-of-treatment outcome status. This nationwide registry analysis may involve correlation of outcomes within geographic clusters. We addressed this using robust standard errors clustered by region of registration, which yielded slightly wider confidence intervals without changing point estimates. Facility-level clustering could not be directly modeled because facility identifiers were not available in the anonymized extract.

## Figures and Tables

**Table 1:** Dynamics of Tuberculosis in the Republic of Kazakhstan by Sex and Cumulative Reduction Relative to 2014 (2014–2023).

Year	Sex		Total, n	Cumulative Reduction vs. 2014 Year, %
	Male, n (%)	Female, n (%)		
2014	6567 (17.3)	9824 (17.5)	16,391	0
2015	5281 (13.9)	8012 (14.3)	13,293	−18.9
2016	4310 (11.4)	6650 (11.9)	10,960	−33.2
2017	4060 (10.7)	5976 (10.7)	10,036	−38.8
2018	3668 (9.7)	5230 (9.3)	8898	−45.7
2019	3452 (9.1)	4787 (8.5)	8239	−49.7
2020	2629 (6.9)	3802 (6.8)	6431	−60.8
2021	2600 (6.8)	3830 (6.8)	6430	−60.8
2022	2741 (7.2)	4018 (7.2)	6759	−58.8
2023	2657 (7.0)	3891 (6.9)	6548	−60.1
Overall	37,965 (100)	56,020 (100)	93,985	−9.7

**Table 2:** Structure of tuberculosis cases by type, sex, age group and year of registration (Kazakhstan, 2014–2023).

Characteristic	New Cases n, (%)	Relapse n, (%)	Treatment Failure n, (%)	Treatment After Interruption n, (%)	Total n, (%)	χ^2^ (df)	*p*-Value
Overall	68,769 (73.2)	23,075 (24.6)	944 (1.0)	1197 (1.3)	93,985 (100)		
Sex						633.8 (3)	<0.001
Male	29,341 (77.3)	8034 (21.2)	333 (0.9)	257 (0.7)	37,965 (100)		
Female	39,428 (70.4)	15,041 (26.8)	611 (1.1)	940 (1.7)	56,020 (100)		
Age group (years)						6630.5 (27)	<0.001
0–14	2952 (97.7)	60 (2.0)	8 (0.3)	2 (0.1)	3022 (100)		
15–24	12,385 (90.8)	1128 (8.3)	96 (0.7)	33 (0.2)	13,642 (100)		
25–34	15,714 (80.5)	3382 (17.3)	202 (1.0)	212(1.1)	19,510 (100)		
35–44	12,964 (70.6)	4819 (26.3)	200 (1.1)	369 (2.0)	18,352 (100)		
45–54	10,388 (65.7)	4919 (31.1)	186 (1.2)	314 (2.0)	15,807 (100)		
55–64	8110 (64.6)	4138 (33.0)	142 (1.1)	158 (1.3)	12,548 (100)		
≥65	6256 (59.6)	4631 (38.4)	110 (1.0)	109 (1.0)	10,763 (100)		
Year of registration						1768.5 (27)	<0.001
2014	10,764 (65.7)	5155 (31.5)	212 (1.3)	260 (1.6)	16,391 (100)		
2017	7392 (73.7)	2335 (23.3)	161 (1.6)	148 (1.5)	10,036 (100)		
2020	5135 (79.8)	1222 (19.0)	17 (0.3)	57 (0.9)	6431 (100)		
2023	5243 (80.1)	1262 (19.3)	2 (<0.0)	41 (0.6)	6548 (100)		

Pearson’s χ^2^ test was used to compare distributions of case types across sex, age groups, and years. Statistical significance was defined as *p* < 0.05.

**Table 3:** Factors associated with unfavorable treatment outcome among tuberculosis patients.

Variable	Category	aOR	95% CI	*p*-Value
Sex (ref. Female)				
Male		1.25	1.17–1.33	<0.001
Age group (years) (ref. (25-34))				
0–14		0.55	0.43–0.70	<0.001
15–24		0.80	0.73–0.88	<0.001
35–44		1.10	1.01–1.20	0.035
45–54		1.25	1.15–1.36	<0.001
55–64		1.45	1.31–1.60	<0.001
65–74		1.65	1.45–1.88	<0.001
≥75		2.10	1.76–2.51	<0.001
Case type (ref. New case)				
Relapse		1.90	1.73–2.08	<0.001
Treatment after interruption		2.60	2.20–3.07	<0.001
Treatment failure category		2.10	1.60–2.76	<0.001
Localization (ref. Pulmonary)				
Extrapulmonary		0.70	0.61–0.80	<0.001
Pulmonary + extrapulmonary		1.40	1.12–1.75	0.003
Smear microscopy (ref. Smear-negative)				
Smear-positive		1.60	1.48–1.73	<0.001
ICD-10 diagnosis (ref. A16)				
A15		1.35	1.23–1.48	<0.001
A17		1.80	0.97–3.35	0.064
A18		0.85	0.74–0.98	0.025
A19		2.05	1.76–2.39	<0.001
Calendar period				
2020–2021		1.15	1.04–1.27	0.006
2022–2023		0.90	0.81–1.00	0.044

Note: 95% confidence intervals and *p*-values were computed using Huber–White robust standard errors clustered by region of registration.

## Data Availability

All available data is presented within the manuscript.

## Supplementary Materials

The following supporting information can be downloaded at https://www.mdpi.com/article/10.3390/tropicalmed11030075/s1, Table S1. Comparison of conventional and clustered inference for the multivariable logistic regression.

## Abbreviations

The following abbreviations are used in this manuscript:

**Abbreviation**	**Definition**
AAPC	Average Annual Percent Change
aOR	Adjusted Odds Ratio
CI	Confidence Interval
COVID-19	Coronavirus Disease 2019
DOTS	Directly Observed Treatment, Short-course
HIV	Human Immunodeficiency Virus
ICD-10	International Classification of Diseases, 10th Revision
MDR-TB	Multidrug-Resistant Tuberculosis
MDR/RR-TB	Multidrug-Resistant/Rifampicin-Resistant Tuberculosis
RR-TB	Rifampicin-Resistant Tuberculosis
SPSS	Statistical Package for the Social Sciences
TB	Tuberculosis
WHO	World Health Organization
χ^2^	Pearson’s Chi-square test
