# Supplementary material for: Trends in Tuberculosis Incidence and Treatment Outcomes in Kazakhstan: A Decade of Observational Data
Source: Trop Med Infect Dis. 2026 Mar 6;11(3):75. doi: 10.3390/tropicalmed11030075 (PMC13030362; doi:10.3390/tropicalmed11030075)
Supplement: Supplementary file 1 [file tropicalmed-11-00075-s001.zip › tropicalmed-4140966-supplementary.pdf]

Supplementary Materials

Supplementary Table S1. Comparison of conventional and clustered inference for the multivariable logistic regression.

Variable	Category	aOR	Conventional 95% CI	Conventional p-value	Cluster-robust 95% CI	Cluster-robust p-value
Sex (ref. Female)	Male	1.25	1.18–1.32	<0.001	1.17–1.33	<0.001
Age group (years) (ref. 25–34)	0–14	0.55	0.45–0.68	<0.001	0.43–0.70	<0.001
Age group (years) (ref. 25–34)	15–24	0.80	0.74–0.87	<0.001	0.73–0.88	<0.001
Age group (years) (ref. 25–34)	35–44	1.10	1.02–1.19	0.01	1.01–1.20	0.035
Age group (years) (ref. 25–34)	45–54	1.25	1.16–1.35	<0.001	1.15–1.36	<0.001
Age group (years) (ref. 25–34)	55–64	1.45	1.33–1.58	<0.001	1.31–1.60	<0.001
Age group (years) (ref. 25–34)	65–74	1.65	1.48–1.85	<0.001	1.45–1.88	<0.001
Age group (years) (ref. 25–34)	≥75	2.10	1.80–2.45	<0.001	1.76–2.51	<0.001
Case type (ref. New case)	Relapse	1.90	1.75–2.05	<0.001	1.73–2.08	<0.001
Case type (ref. New case)	Treatment after interruption	2.60	2.25–3.00	<0.001	2.20–3.07	<0.001
Case type (ref. New case)	Treatment failure category	2.10	1.65–2.65	<0.001	1.60–2.76	<0.001
Localization (ref. Pulmonary)	Extrapulmonary	0.70	0.62–0.79	<0.001	0.61–0.80	<0.001
Localization (ref. Pulmonary)	Pulmonary + extrapulmonary	1.40	1.15–1.70	<0.001	1.12–1.75	0.003
Smear microscopy (ref. Smear-negative)	Smear-positive	1.60	1.50–1.72	<0.001	1.48–1.73	<0.001
ICD-10 diagnosis (ref. A16)	A15	1.35	1.25–1.46	<0.001	1.23–1.48	<0.001
ICD-10 diagnosis (ref. A16)	A17	1.80	1.05–3.10	0.03	0.97–3.35	0.064
ICD-10 diagnosis (ref. A16)	A18	0.85	0.75–0.96	0.01	0.74–0.98	0.025
ICD-10 diagnosis (ref. A16)	A19	2.05	1.80–2.35	<0.001	1.76–2.39	<0.001
Calendar period (ref. 2014–2016)	2020–2021	1.15	1.05–1.25	0.002	1.04–1.27	0.006
Calendar period (ref. 2014–2016)	2022–2023	0.90	0.82–0.98	0.01	0.81–1.00	0.044
